# Microbiota Dysbiosis: A Key Modulator in Preeclampsia Pathogenesis and Its Therapeutic Potential

**DOI:** 10.3390/microorganisms13020245

**Published:** 2025-01-23

**Authors:** Johnatan Torres-Torres, Jorge Alberto Basurto-Serrano, Zaira Alexi Camacho-Martinez, Francisco Rafael Guadarrama-Sanchez, Irma Eloisa Monroy-Muñoz, Javier Perez-Duran, Juan Mario Solis-Paredes, Raigam Martinez-Portilla, Salvador Espino-y-Sosa, Andrea Ramirez-Gonzalez, Rodrigo Guadarrama-Mora, Lourdes Rojas-Zepeda

**Affiliations:** 1 Department of Reproductive and Perinatal Health Research, Instituto Nacional de Perinatología Isidro Espinosa de los Reyes, Mexico City 11000, Mexico; irmae4901@gmail.com (I.E.M.-M.); djavier40@gmail.com (J.P.-D.); juan.mario.sp@gmail.com (J.M.S.-P.); raifet@hotmail.com (R.M.-P.); salvadorespino@gmail.com (S.E.-y.-S.); rodguamor94@gmail.com (R.G.-M.); 2 Obstetric and Gynecology Department, Hospital General de México Dr. Eduardo Liceaga, Mexico City 06720, Mexico; zay.alexi9@gmail.com (Z.A.C.-M.); a.rmzglz@outlook.es (A.R.-G.); 3 Maternal-Fetal Department, Instituto Materno Infantil del Estado de Mexico, Toluca 50170, Mexico; betobasurto@gmail.com; 4 Obstetric and Gynecology Department, Hospital San Jose, Queretaro 76187, Mexico; fcoguadas@gmail.com

**Keywords:** preeclampsia pathogenesis, microbiota dysbiosis, gut and vaginal microbiota, immune regulation and inflammation, probiotics and microbiota interventions

## Abstract

Preeclampsia is a leading cause of maternal and perinatal morbidity and mortality worldwide. Emerging evidence implicates gut and vaginal microbiota dysbiosis in preeclampsia pathogenesis through its roles in immune regulation, inflammation, and placental function. This review explores the mechanisms linking microbiota alterations to preeclampsia and evaluates the therapeutic potential of microbiota-targeted interventions. A systematic search using MeSH terms related to “preeclampsia”, “microbiota”, and “dysbiosis” identified studies on microbiota and preeclampsia pathophysiology. Data extraction focused on microbial alterations and mechanistic insights. Gut dysbiosis, characterized by reduced beneficial bacteria and short-chain fatty acid production, weakens the intestinal barrier, exacerbates systemic inflammation, and impairs placental development. Vaginal dysbiosis, marked by reduced *Lactobacillus* species, promotes local inflammation, increasing placental dysfunction risk. Therapeutic strategies, including probiotics, prebiotics, and dietary modifications, show promise in restoring microbial balance and mitigating preeclampsia risk. Microbiota dysbiosis significantly contributes to preeclampsia pathogenesis through inflammation, endothelial dysfunction, and placental impairment. Interventions targeting microbial balance, such as probiotics and dietary modifications, show promise for prevention, but further research and large-scale trials are essential to validate their efficacy and safety.

## 1. Introduction

Preeclampsia (PE) is a leading cause of maternal and perinatal morbidity and mortality worldwide, affecting 3–5% of pregnancies [1]. This condition, characterized by hypertension and multisystemic damage, poses significant risks to maternal and fetal health [2,3]. While its precise triggers remain unclear, placental dysfunction, exaggerated inflammation, and defective vascular remodeling are central to its pathophysiology [4].

Recent research highlights the microbiota—the trillions of microorganisms residing in the human body—as a novel focus for understanding PE risk [5,6]. These microbial communities are critical for immunological, metabolic, and inflammatory homeostasis [7]. The gut and vaginal microbiota play key roles in maintaining pregnancy health by modulating immune responses and preventing inflammation. Dysbiosis, or microbial imbalance—characterized by a reduction in microbial diversity, the proliferation of harmful bacteria, and a decrease in beneficial microorganisms—has been linked to inflammatory, metabolic, and obstetric complications [5,8]. During pregnancy, the gut microbiota influences immune and metabolic processes, producing short-chain fatty acids (SCFAs) that support intestinal barrier integrity and suppress inflammation [9,10]. Dysbiosis reduces microbial diversity, fostering chronic inflammation and increasing PE risk [5]. Similarly, the vaginal microbiota, typically dominated by *Lactobacillus* species, prevents pathogen invasion and maintains local homeostasis [11]. Vaginal dysbiosis, characterized by a loss of *Lactobacillus* and an overgrowth of anaerobic bacteria such as *Gardnerella vaginalis* and *Prevotella*, can compromise this barrier, allowing infections to ascend to the uterus and trigger localized and systemic inflammation. This inflammation may disrupt placental function, impair trophoblast invasion, and contribute to PE development [12]. Emerging evidence suggests that microbiota–placenta interactions, including “gut–placenta” and “vaginal microbiota–placenta” axes, play a significant role in modulating placental health [13]. These axes highlight the microbiota’s potential as a target for interventions aimed at improving maternal-fetal outcomes.

This review synthesizes current evidence on the microbiota’s role in PE development, examining biological mechanisms and potential therapeutic applications. It also identifies research gaps, such as the need to clarify causal links between dysbiosis and PE and to evaluate the effectiveness of microbiota-based therapies in prevention strategies.

## 2. Materials and Methods

### 2.1. Eligibility Criteria

We performed a systematic search utilizing specific MeSH terms related to both preeclampsia and microbiota, such as “preeclampsia”, “microbiota”, “intestinal microbiota”, “vaginal microbiota”, “dysbiosis”, “pathophysiology”, “inflammation”, and “immune response”. The detailed search strategy, along with specific query syntaxes, is outlined in Table S1.

Studies included in this review were original research articles from both basic and clinical science, as well as systematic reviews and narrative reviews that investigated the relationship between microbiota (intestinal and vaginal) and the risk or pathophysiology of preeclampsia. Both observational and interventional studies were eligible for inclusion. This review adhered to the PRISMA guidelines for systematic reviews (Figure S1).

### 2.2. Study Selection

Abstracts retrieved through the search strategy were independently screened by two reviewers (J.T.T. and J.A.B.S.). The initial screening, based on titles and abstracts, was performed blind to authorship and institutional affiliations to minimize selection bias. Articles deemed potentially relevant were selected for full-text review. In the second phase, these full texts were thoroughly examined to ensure they met the predetermined inclusion criteria. Any disagreements between reviewers regarding study eligibility were resolved through discussion and, if necessary, consultation with a third reviewer.

### 2.3. Data Extraction and Analysis

Data were extracted from the included studies using a standardized data collection form designed to capture key study characteristics such as author, publication year, country of origin, study design, and main outcomes related to microbiota and preeclampsia. This form also gathered specific findings regarding microbiota composition (intestinal or vaginal), its alterations (dysbiosis), and potential mechanistic links with preeclampsia pathogenesis.

The extracted data were then systematically analyzed and categorized into relevant sections for this review. Major findings were emphasized, and cross-links were drawn between different studies that explored the interplay between microbiota and preeclampsia. Additionally, we highlighted potential therapeutic implications based on these interactions. This structured approach allowed for a comprehensive synthesis of the existing literature, providing a detailed overview of the current understanding of how microbiota may influence the development and progression of preeclampsia (Table S2).

## 3. Results

The results of this review highlight the significant roles that microbiota, particularly gut and vaginal and possibly placental microbiota, play in the development and progression of PE. Each microbial environment interacts with maternal immune regulation, inflammation, and placental function, contributing to the overall pathophysiology of the condition (Table 1).

### 3.1. Gut Microbiota and Preeclampsia

The gut microbiota regulates metabolic, immunological adaptations, and inflammatory processes during pregnancy and promotes fetal tolerance [14,15]. Several studies have reported significant gut dysbiosis in preeclamptic women, characterized by a reduction in microbial diversity and a reduction of anti-inflammatory species such as *Lactobacillus* and *Bifidobacterium* [16,17]. These bacteria are essential for producing short-chain fatty acids (SCFAs) such as butyrate, propionate, and acetate, which are crucial in maintaining immune homeostasis and gut barrier integrity. SCFAs mediate their effects through multiple molecular pathways. They act by binding to G-protein-coupled receptors (GPR41 and GPR43) on intestinal epithelial cells and immune cells, activating anti-inflammatory signaling pathways that suppress the nuclear factor kappa B (NF-κB) pathway, a key driver of inflammation. SCFAs also enhance intestinal barrier integrity by increasing the expression of tight junction proteins (e.g., occludin and claudins) and stimulating mucin production, which strengthens the protective mucus layer [18]. Additionally, butyrate serves as an energy source for colonocytes, further supporting epithelial integrity and reducing intestinal permeability.

In the context of gut dysbiosis, reduced SCFA production compromises these protective mechanisms, leading to weakened gut barrier function and increased translocation of bacterial endotoxins such as lipopolysaccharides (LPS) into the bloodstream [9]. LPS, in turn, binds to Toll-like receptor 4 (TLR4) on innate immune cells, triggering the release of pro-inflammatory cytokines, including tumor necrosis factor-alpha (TNF-α) and interleukin-6 (IL-6) [19]. These cytokines exacerbate systemic inflammation and endothelial dysfunction, which are central to the pathogenesis of preeclampsia. Endothelial dysfunction impairs spiral artery remodeling, resulting in reduced placental perfusion and hypoxia. Hypoxia then stimulates the release of anti-angiogenic factors, such as soluble Fms-like tyrosine kinase-1 (sFlt-1), which further aggravates vascular dysfunction and perpetuates a cycle of inflammation, vasoconstriction, and multiorgan damage characteristic of preeclampsia [15]. Thus, gut dysbiosis contributes to perpetuates a cycle of inflammation, vasoconstriction, and multiorgan damage that underpins the systemic pathology of PE [18].

### 3.2. Gut–Placenta Microbiota Axis

**Figure 1:**
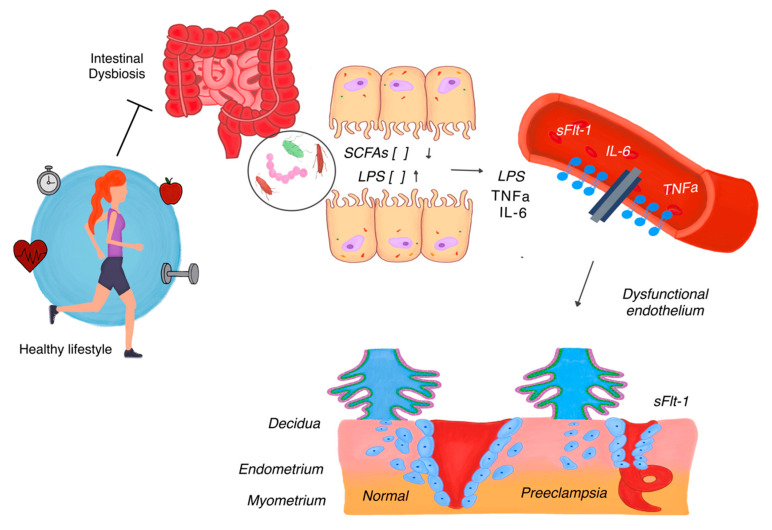
The role of gut microbiota in preeclampsia pathogenesis. A healthy lifestyle, including exercise and a balanced diet, promotes microbial diversity and the production of short-chain fatty acids (SCFAs), which maintain the intestinal barrier and modulate inflammation. In contrast, intestinal dysbiosis leads to reduced SCFA production and increased intestinal permeability, allowing lipopolysaccharides (LPS) to enter the circulation. LPS triggers systemic inflammation by promoting the release of pro-inflammatory cytokines, such as tumor necrosis factor-alpha (TNF-α) and interleukin-6 (IL-6), which contribute to endothelial dysfunction. The dysfunctional endothelium, coupled with elevated anti-angiogenic factors such as soluble Fms-like tyrosine kinase-1 (sFlt-1), impairs placental vascular remodeling and promotes hypoxia, perpetuating the cycle of inflammation and preeclampsia pathophysiology.

The gut–placenta microbiota axis represents a critical interface where the maternal microbiota interacts with placental function (Figure 1). Gut dysbiosis in PE has been shown to affect placental health through altered microbial metabolites, systemic inflammation, and increased intestinal permeability, which allows bacterial components such as LPS to translocate/move into the bloodstream. These microbial components activate maternal immune pathways via Toll-like receptor 4 (TLR-4), which is expressed on trophoblastic cells in the placenta [20].

TLR-4 activation triggers an inflammatory cascade within the placenta, leading to the production of cytokines such as TNF-α and IL-6, a reduction in IL-10 expression, and dysregulation of macrophages with a predominance of the M1 phenotype. These cytokines disrupt placental vascular function, impairing nutrient and gas exchange between mother and fetus, and contributing to fetal hypoxia [21]. Fetal hypoxia further stimulates the release of pro-inflammatory and anti-angiogenic factors into the maternal circulation, exacerbating the inflammatory condition characteristic of preeclampsia [22].

Additionally, the reduced availability of SCFAs due to gut dysbiosis enhances immune reactivity to inflammatory stimuli, creating a feedback loop of chronic inflammation that exacerbates endothelial and placental dysfunction [23].

### 3.3. Vaginal Microbiota and Preeclampsia Risk

In a healthy state, the vaginal microbiota is predominantly composed of *Lactobacillus* species, which maintain an acidic environment (pH < 4.5) by producing lactic acid [24]. This acidic environment inhibits the growth of pathogenic microorganisms and preserves the integrity of the mucosal barrier, serving as a first line of defense against infections [14]. However, in preeclamptic women, a significant shift in the vaginal microbiota has been observed. This dysbiosis is characterized by a reduction in *Lactobacillus* and an overgrowth of anaerobic bacteria such as *Gardnerella vaginalis*, *Atopobium vaginae*, and *Prevotella* [8] creating conditions conducive to pathogenic invasion and inflammation.

The proliferation of these pathogens compromises the mucosal integrity, allowing the activation of pattern recognition receptors (PRRs) such as Toll-like receptors (TLRs) on vaginal epithelial cells. This leads to the release of pro-inflammatory cytokines, such as interleukin-1β (IL-1β) and TNF-α, further increasing local inflammation [12]. This localized inflammatory response increases the risk of ascending infections, which can reach the uterine cavity and placenta, causing intrauterine inflammation and damage to the decidua.

**Figure 2:**
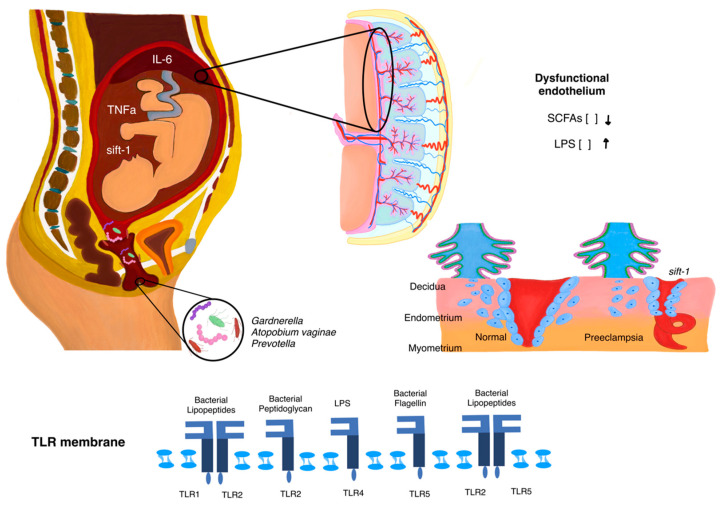
Mechanistic role of vaginal microbiota in preeclampsia development.

The inflammatory state in the reproductive tract can spread systemically, activating immune responses in both the placental and maternal circulatory systems. At the placental interface, chronic immune activation interferes with the invasion of trophoblasts and the remodeling of spiral arteries—both processes critical for ensuring adequate blood flow to the placenta [25]. The resulting impaired vascular development contributes to placental insufficiency and hypoxia, which are key drivers of PE pathogenesis. Thus, vaginal dysbiosis not only facilitates pathogenic entry but also perpetuates a systemic inflammatory state that affects placental function and maternal vascular integrity (Figure 2).

In a healthy state, the vaginal microbiota is dominated by *Lactobacillus* species, which create an acidic environment to inhibit pathogen overgrowth and maintain mucosal integrity. Vaginal dysbiosis, characterized by the reduction of *Lactobacillus* and the overgrowth of anaerobic bacteria such as *Gardnerella vaginalis*, *Atopobium vaginae*, and *Prevotella*, disrupts the mucosal barrier and promotes local inflammation. Bacterial components, such as lipopolysaccharides (LPS), interact with Toll-like receptors (TLRs) on epithelial cells, triggering inflammatory pathways and the release of pro-inflammatory cytokines, including tumor necrosis factor-alpha (TNF-α) and interleukin-6 (IL-6). This inflammatory response extends to the uterus, impairing placental function and spiral artery remodeling. The dysfunctional endothelium contributes to increased release of anti-angiogenic factors such as soluble Fms-like tyrosine kinase-1 (sFlt-1), leading to placental insufficiency, hypoxia, and the systemic pathophysiology observed in preeclampsia. The TLR pathway is highlighted, showcasing the molecular recognition of bacterial components that drive the inflammatory cascade.

### 3.4. Placental Microbiota and Preeclampsia

Recent research challenges the long-held belief that the placenta is a sterile environment. The detection of microbial DNA in placental tissue has sparked hypotheses that the placenta may harbor a unique microbiota, potentially influencing pregnancy outcomes. Studies have reported the presence of bacterial DNA from genera such as *Firmicutes*, *Proteobacteria*, and *Bacteroidetes*, which suggests the possibility of microbial communities in the placenta. However, whether these communities are viable and metabolically active or represent contamination remains a subject of debate [26]. Advances in sequencing technologies, such as metagenomics and transcriptomics, have provided tools to differentiate true microbial signatures from potential contaminants, yet the evidence remains inconclusive.

In the context of PE, potential placental dysbiosis could disrupt critical processes involved in placental development and function. For instance, altered microbial activity may influence immune interactions within the placenta. Placental immune cells, including macrophages and trophoblasts, are key players in maintaining local immune tolerance while modulating inflammatory responses necessary for vascular remodeling. Microbial components, such as LPS or bacterial DNA, can activate TLRs on these cells, potentially shifting the balance toward a pro-inflammatory state. This could impair trophoblast invasion, a process critical for proper spiral artery remodeling and adequate placental perfusion [27,28]. Furthermore, the placental microbiota may interact with angiogenic and anti-angiogenic pathways. For example, microbial dysbiosis might exacerbate the release of anti-angiogenic factors such as sFlt-1, which are already elevated in PE. By disrupting trophoblast-endothelial cell interactions, this dysregulation may impair nutrient and oxygen exchange between mother and fetus, contributing to placental insufficiency and fetal hypoxia [28]. Hypoxia, in turn, can amplify inflammatory responses within the placenta, perpetuating a cycle of vascular dysfunction and systemic inflammation characteristic of PE.

While the potential role of the placental microbiota opens new research avenues, it is crucial to acknowledge the limitations of current evidence. Most studies have focused on microbial DNA detection rather than functional activity, and the small bacterial loads reported in placental tissues make distinguishing true colonization from contamination challenging. Emerging evidence suggests that microbial signals may reflect systemic changes in maternal microbiota rather than a distinct placental microbiome [26]. For instance, microbial metabolites such as short-chain fatty acids (SCFAs) produced in the gut can influence systemic inflammation and placental development, indirectly linking maternal gut dysbiosis to placental dysfunction observed in PE [27] (Table 2).

Robust studies are needed to determine whether the placental microbiota plays a causative role in pregnancy complications or serves as a biomarker of maternal and fetal health. Future research employing rigorous controls, improved sequencing methodologies, and functional assays will be critical to elucidate the true nature and functional relevance of the placental microbiota in PE. Until then, the hypothesis remains an intriguing yet unresolved aspect of pregnancy research.

### 3.5. Impact of the Microbiota on Maternal Immune System Function

Beyond localized effects, alterations in the gut and vaginal microbiota significantly impact the maternal immune system’s ability to maintain the delicate balance between immune tolerance toward the fetus and the need to protect against infections. In a healthy pregnancy, immune adaptations prevent maternal rejection of fetal cells while modulating inflammatory responses [22]. In PE, dysbiosis leads to immune dysregulation, with overactivation of pro-inflammatory pathways and reduced anti-inflammatory signaling.

Key immune cells, particularly T-regulatory cells, are essential for maintaining fetal tolerance and are influenced by microbiota-derived metabolites, particularly SCFAs. Reduced SCFA levels, as seen in dysbiosis, impair the function of T-regulatory cells, leading to an exaggerated inflammatory response [29,30]. This heightened immune reactivity exacerbates endothelial dysfunction and placental damage, further fueling the progression of preeclampsia.

### 3.6. External Factors Modulating Microbiota and Preeclampsia Risk

The maternal microbiota is shaped by endogenous factors and external influences such as diet, antibiotic use, obesity, and stress referred to as the exposome. These factors can either support a healthy microbiota or drive dysbiosis, potentially influencing the risk of PE (Table 3).

Diet is a primary modifiable factor; a fiber-rich diet promotes beneficial microbial populations that produce anti-inflammatory metabolites, while high-fat or ultra-processed foods can foster an imbalance in the microbiota, leading to dysbiosis and increasing systemic inflammation [22]. Although sometimes necessary, antibiotic use during pregnancy can have long-lasting effects on microbiota composition, disrupting the protective bacterial communities and potentially heightening the risk of inflammatory complications [26]. Similarly, obesity has been associated with reduced microbial diversity and a pro-inflammatory microbiota profile, both of which are linked to an increased risk of metabolic and hypertensive disorders, including PE [22].

The exposome also influences microbiota composition. For example, stress-induced changes affect the gut microbiota, which may enhance intestinal permeability and lead to systemic inflammation, while a sedentary lifestyle can further exacerbate these effects [31]. These factors highlight the need for a holistic approach to maternal health, wherein modifiable lifestyle interventions, including dietary and stress management strategies, could play a crucial role in maintaining a balanced microbiota and reducing PE risk.

### 3.7. Interventions Targeting Microbiota to Prevent Preeclampsia

Given the microbiota’s role in shaping immune responses and influencing inflammation, interventions aimed at modulating microbial balance offer a potential therapeutic avenue for preventing preeclampsia [32]. Probiotics, prebiotics, and dietary modifications have been investigated for their capacity to restore microbial equilibrium, enhance the production of beneficial metabolites such as SCFAs, and reinforce the integrity of mucosal barriers [26].

Probiotic supplementation, particularly with strains like *Lactobacillus* and *Bifidobacterium*, has shown promise in clinical studies. These probiotics are known to promote microbial diversity and may reduce inflammation by modulating gut and vaginal flora. By promoting an anti-inflammatory environment, probiotics could potentially reduce the risk of PE. Prebiotics, such as dietary fibers, further stimulate the growth of these beneficial bacteria and support the production of SCFAs, which are crucial for maintaining gut health [33].

In addition to probiotics and prebiotics, specific dietary patterns, such as the Mediterranean diet and diets rich in fiber, have emerged as non-pharmacological strategies to prevent preeclampsia by modulating the microbiota. The Mediterranean diet, characterized by high consumption of fruits, vegetables, whole grains, legumes, and unsaturated fats such as olive oil, has been associated with increased microbial diversity and reduced systemic inflammation. This diet promotes the production of SCFAs, which enhances the integrity of the intestinal barrier, decreases the translocation of bacterial endotoxins, and mitigates the release of pro-inflammatory cytokines like TNF-α and IL-6 [10,26,33]. Similarly, fiber-rich diets have been shown to support the growth of beneficial bacterial species, including *Lactobacillus* and *Bifidobacterium*, and improve vascular function through anti-inflammatory mechanisms [32] (Table 4).

While preliminary findings are promising, the application of these interventions in routine obstetric care remains limited by the lack of large-scale clinical trials. Future research will need to confirm the effectiveness and safety of microbiota-targeted therapies in pregnant populations, particularly those at high risk of developing PE. Specifically, randomized controlled trials are needed to evaluate the impact of dietary modifications, probiotics, and prebiotics on maternal and fetal outcomes, including inflammation, endothelial function, and pregnancy complications. Additionally, research should explore personalized strategies that consider genetic predispositions, lifestyle factors, and regional dietary patterns to optimize microbiota modulation. By addressing these gaps, the field can pave the way for innovative, evidence-based interventions that offer non-invasive and accessible solutions to mitigate preeclampsia risk globally.

## 4. Discussion

### 4.1. Principal Findings

This analysis emphasizes the critical role of gut, placental, and vaginal microbiota in the pathogenesis of PE, revealing that dysbiosis may trigger systemic inflammatory responses and endothelial dysfunction, which are central factors in the development of this condition. The findings suggest that microbial balance is essential for immune regulation and placental function, with microbial alterations directly linked to maternal-fetal complications observed in PE. Thus, the microbiota emerges not only as a risk factor but also as a potential therapeutic target to reduce the incidence and severity of this disease.

### 4.2. Clinical Implication

These findings highlight the significant potential of microbiota-based interventions in preventing and managing PE. Non-invasive strategies, such as probiotics, prebiotics, and dietary modifications, present a promising approach to restoring microbial balance, reducing inflammation, and addressing risk factors in high-risk women. Integrating these interventions into prenatal care could be particularly beneficial in resource-limited settings, offering accessible tools to reduce PE incidence and severity. However, large-scale clinical trials are necessary to confirm their efficacy and safety across diverse populations and clinical contexts.

Specific probiotic strains, such as *Lactobacillus rhamnosus* HN001, have shown benefits during pregnancy. For instance, a clinical trial in New Zealand demonstrated a significant reduction in gestational diabetes incidence among women who received this probiotic, attributed to improved gut microbiota modulation, enhanced metabolic homeostasis, and reduced systemic inflammation [34]. These mechanisms are relevant to PE, as shared factors like inflammation and metabolic stress contribute to its pathogenesis. By mitigating systemic inflammation and reducing endotoxin-mediated endothelial dysfunction—a key mechanism in PE—probiotic interventions like *Lactobacillus rhamnosus* HN001 may have broader preventive applications for hypertensive disorders of pregnancy.

Dietary fiber intake also plays a critical role in mitigating PE risk. A prospective study found that women who consumed high levels of dietary fiber (≥21.2 g/day) in early pregnancy had a 67% lower risk of developing PE compared to those with lower fiber intake [35]. This protective effect is likely mediated through reductions in dyslipidemia, blood pressure, and inflammation—key contributors to PE pathogenesis. Similarly, a randomized trial in China reported that fiber supplementation (12 g twice daily) in overweight or obese women during mid-pregnancy significantly reduced the risk of gestational diabetes and preterm birth [36]. Although focused on diabetes, these findings suggest that dietary fiber improves metabolic health, which is directly relevant to PE prevention. Additionally, broader dietary patterns, such as a diet rich in fruits, vegetables (≥400 g/day), plant-based foods, and unsaturated fats, while limiting high-fat, high-sugar, and high-salt foods, have been linked to reduced PE risk. Such diets promote microbial diversity, enhance short-chain fatty acid production, and reduce systemic inflammation, further supporting vascular and metabolic health during pregnancy. Adequate fiber intake (25–30 g/day) is especially recommended to amplify these benefits by improving lipid profiles, lowering blood pressure, and curbing inflammatory processes associated with PE [37].

### 4.3. Research Implication

This review opens several critical research avenues. First, establishing a direct causal relationship between dysbiosis and preeclampsia requires longitudinal and experimental studies. Additionally, it is crucial to characterize the specific effects of different microbial compositions on the maternal immune and vascular systems. Future research should also explore the efficacy of personalized microbiome-based interventions, considering factors such as genetics and lifestyle, which modulate microbial composition and response. Finally, studying the placental microbiome, although still debated, may offer novel insights into the direct impact of microorganisms on placental function and the development of obstetric complications.

### 4.4. Strength and Limitations

This analysis is strengthened by its comprehensive approach and integration of both basic and clinical studies, providing a broad perspective on the microbiota–preeclampsia relationship. The inclusion of gut, vaginal, and potentially placental microbiota enables a robust understanding of underlying mechanisms and potential clinical applications. However, limitations exist, current studies exhibit significant heterogeneity in design, methodologies, and outcomes, making it difficult to draw definitive conclusions. Most available evidence is derived from observational studies or small-scale trials, which lack the rigor and control of randomized controlled trials (RCTs). Furthermore, key gaps in knowledge remain unresolved, such as the identification of optimal probiotic or prebiotic strains, the appropriate dosages, and the timing and duration of interventions needed to achieve therapeutic benefits. While the existing data suggest a significant association between dysbiosis and preeclampsia, the lack of longitudinal and mechanistic studies precludes the establishment of a direct causal relationship. These limitations hinder the immediate translation of findings into clinical practice. To address these challenges, large-scale, well-designed RCTs are urgently needed to validate the safety and efficacy of microbiota-based interventions, standardize intervention protocols, and explore how factors such as genetics, lifestyle, and diet influence individual responses to these therapies.

## 5. Conclusions

This review underscores the critical role of microbiota dysbiosis in the pathogenesis of preeclampsia, highlighting its contributions to systemic inflammation, endothelial dysfunction, and placental impairment. Targeted interventions, including probiotics, prebiotics, and dietary modifications, hold promise for reducing PE risk by restoring microbial balance and improving immune regulation.

While these findings advance our understanding of preeclampsia’s biological mechanisms, significant gaps remain. Future research should focus on identifying the most effective probiotic strains, optimizing intervention timing, and tailoring approaches to individual risk profiles. Additionally, large-scale clinical trials are needed to validate the safety and efficacy of microbiota-targeted therapies in diverse populations. Addressing these gaps will pave the way for innovative, non-invasive strategies to improve maternal and fetal outcomes globally.

## Figures and Tables

**Table 1:** Microbiota dysbiosis and its impact on preeclampsia.

Aspect	Description	Observed Alterations	Effect	Key Factor
Gut Microbiota	Refers to the collection of microorganisms residing in the intestine, which are critical for immune regulation, metabolic processes, and inflammation during pregnancy.	Decrease in beneficial bacteria (*Lactobacillus*, *Bifidobacterium*) responsible for producing SCFAs. Increase in pro-inflammatory bacteria such as *Escherichia coli* and *Enterococcus faecalis*.	Reduced SCFA production weakens the gut barrier, leading to increased intestinal permeability. Bacterial endotoxins (e.g., LPS) enter the bloodstream, triggering systemic inflammation and immune system activation. Systemic inflammation exacerbates endothelial dysfunction, a hallmark of preeclampsia.	SCFAs (butyrate, propionate), gut permeability, LPS.
Vaginal Microbiota	Refers to microorganisms in the vaginal tract, which maintain local homeostasis, prevent infections, and support immune regulation.	Reduction of *Lactobacillus* spp., which maintains an acidic pH. Overgrowth of anaerobic bacteria such as *Gardnerella vaginalis* and *Prevotella*.	Loss of vaginal acidity allows pathogenic bacteria to thrive, causing local inflammation. Inflammation increases the risk of ascending infections, which can reach the uterus and placenta. Ascending infections impair placental function and disrupt spiral artery remodeling, leading to placental insufficiency and fetal hypoxia.	*Lactobacillus* spp., *Gardnerella vaginalis*, *Prevotella*, local pH balance, ascending infections.
Placental Microbiota	Refers to emerging evidence suggesting the placenta may host its own microbiota, although its existence and role are debated.	Detection of microbial DNA from genera such as *Firmicutes*, *Proteobacteria*, and *Bacteroidetes*. Evidence remains inconclusive about whether these microbes are viable or contaminants.	Microbial DNA may interfere with critical placental functions such as trophoblast invasion and angiogenesis. Disruption in placental immune signaling contributes to placental insufficiency, reduced nutrient exchange, and exacerbated maternal inflammation.	Microbial DNA, inflammatory signals, trophoblast invasion, angiogenesis disruption.
Interventions Targeting Microbiota	Strategies aimed at restoring microbial balance using dietary or pharmacological approaches to improve maternal and fetal health outcomes.	Use of probiotics to replenish beneficial bacteria such as *Lactobacillus* and *Bifidobacterium*. Use of prebiotics such as dietary fibers to stimulate the growth of beneficial bacteria.	Probiotics and prebiotics enhance SCFA production, reduce systemic inflammation, and strengthen the intestinal and vaginal barriers. These interventions may mitigate risk factors for preeclampsia by restoring microbial homeostasis.	Probiotics [*Lactobacillus*, *Bifidobacterium*], prebiotics [dietary fiber], SCFAs production.
External Factors Modulating Microbiota	Exogenous factors that influence microbiota composition during pregnancy and may contribute to dysbiosis.	Diet: high-fiber diets support beneficial bacteria, while high-fat or processed food diets promote dysbiosis. Antibiotics: alter the composition and diversity of protective microbiota. Obesity: associated with a pro-inflammatory microbiota profile and reduced microbial diversity.	High-fiber diets promote microbial diversity and SCFA production, reducing inflammation. Antibiotics can disrupt microbial communities, increasing susceptibility to inflammatory complications. Obesity fosters a dysbiotic, pro-inflammatory microbiota that exacerbates systemic inflammation and endothelial dysfunction.	Fiber, processed foods, antibiotics, obesity, metabolic inflammation.

SCFAs: short-chain fatty acids; LPS: lipopolysaccharides.

**Table 2:** Potential roles of placental microbiota in preeclampsia.

Aspect	Description	Implications in Preeclampsia
Microbiota Composition	Detection of bacterial DNA from *Firmicutes, Proteobacteria*, and *Bacteroidetes* in placental tissue.	May represent viable microbial communities or contamination; functional activity and relevance remain uncertain. Advances in sequencing, such as metagenomics and transcriptomics, aim to distinguish true colonization from contamination.
Potential Mechanisms	Activation of TLRs by microbial components such as LPS or bacterial DNA.	Triggers pro-inflammatory cascades, shifting the balance toward inflammation within the placenta. Impairs trophoblast invasion and angiogenesis, contributing to vascular dysfunction and placental insufficiency.
	Dysregulated release of pro-inflammatory cytokines, including TNF-α and IL-6.	Increases endothelial dysfunction and systemic inflammation, leading to impaired spiral artery remodeling and reduced placental perfusion.
	Exacerbation of anti-angiogenic factors such as sFlt-1.	Disrupts trophoblast-endothelial interactions, impairing nutrient and oxygen exchange between mother and fetus. Amplifies placental insufficiency and hypoxia.
Effects on the Placenta	Disruption of immune interactions within the placenta.	Alters local immune tolerance and inflammatory signaling, further compromising vascular remodeling and placental function.
	Reduction in trophoblast invasion and spiral artery remodeling.	Leads to placental hypoxia, fetal nutrient restriction, and exacerbation of systemic inflammation.
Current Knowledge Status	Evidence suggests microbial signals may reflect systemic maternal changes rather than a distinct placental microbiome. Small bacterial loads complicate differentiation between true colonization and contamination.	Limited data on functional microbial activity; unclear whether alterations are causative in preeclampsia or secondary to systemic changes. Further studies are essential to clarify microbial viability and functional relevance.
Future Research Directions	Use of metagenomics, transcriptomics, and advanced functional assays to characterize microbial communities and differentiate contamination from true colonization.	Identification of functional microbial components and their role in inflammation, angiogenesis, and vascular remodeling. Experimental models to explore microbiota–host interactions and their impact on placental health and preeclampsia.

TLRs: Toll-like receptors; LPS: lipopolysaccharides; TNF-α: Tumor necrosis factor-alpha; IL-6: Interleukin-6.

**Table 3:** External factors and microbiota’s role in preeclampsia.

External Factor	Description	Effect on Microbiota	Implications for Preeclampsia
Diet	High-fat or ultra-processed foods promote dysbiosis.	Reduces microbial diversity and short-chain fatty acid production.	Imbalanced diets increase systemic inflammation and the risk of preeclampsia.
Antibiotic Use	Disrupts protective bacterial communities; sometimes necessary but with potential long-term effects.	Reduces microbial diversity.	Heightens susceptibility to inflammatory complications.
Obesity	Associated with reduced microbial diversity and a pro-inflammatory microbiota profile.	Creates a pro-inflammatory microbiota with decreased diversity.	Increases the risk of metabolic and hypertensive disorders, including preeclampsia.
Stress	Alters gut microbiota, increasing intestinal permeability and systemic inflammation.	Increases intestinal permeability and systemic inflammation.	Contributes to endothelial dysfunction and other preeclampsia-related complications.
Sedentary Lifestyle	Aggravates the negative effects of other factors.	Reduces microbiota resilience to harmful factors.	Potentially increases inflammation and metabolic imbalance, predisposing to preeclampsia.

**Table 4:** Microbiota-targeted interventions and their potential benefits in preeclampsia.

Intervention	Mechanism of Action	Potential Benefits in Preeclampsia
Probiotics	Contain beneficial strains like *Lactobacillus* and *Bifidobacterium*. Enhance microbial diversity and modulate gut and vaginal flora.	Reduce systemic inflammation. Strengthen intestinal and vaginal mucosal barriers. Lower pro-inflammatory cytokines (e.g., TNF-α, IL-6).
Prebiotics	Dietary fibers that stimulate the growth of beneficial bacteria and enhance SCFA production.	Support gut barrier integrity. Promote anti-inflammatory pathways. Decrease intestinal permeability and endotoxin translocation.
Mediterranean Diet	Emphasizes high intake of fruits, vegetables, whole grains, legumes, and unsaturated fats (e.g., olive oil).	Increase microbial diversity. Boost SCFAs production. Mitigate systemic inflammation and endothelial dysfunction.
Fiber-Rich Diets	Diets with elevated fiber intake to support the growth of beneficial bacterial species.	Promote *Lactobacillus* and *Bifidobacterium* proliferation. Improve vascular function. Reduce inflammation and oxidative stress.

SCFAs: short-chain fatty acids; TNF-α: Tumor necrosis factor-alpha; IL-6: Interleukin-6.

## Data Availability

The original contributions presented in the study are included in the article/Supplementary Material, further inquiries can be directed to the corresponding authors.

## Supplementary Materials

The following supporting information can be downloaded at: https://www.mdpi.com/article/10.3390/microorganisms13020245/s1, Table S1: Search Strategies; Figure S1: PRISMA flow diagram; Table S2: Summary of included studies.
