# Supplementary material for: Microbiota Dysbiosis: A Key Modulator in Preeclampsia Pathogenesis and Its Therapeutic Potential
Source: Microorganisms. 2025 Jan 23;13(2):245. doi: 10.3390/microorganisms13020245 (PMC11857279; doi:10.3390/microorganisms13020245)
Supplement: Supplementary file 1 [file microorganisms-13-00245-s001.zip › Figure S1. PRISMA microbiota.pdf]

Figure S1. PRISMA flow diagram

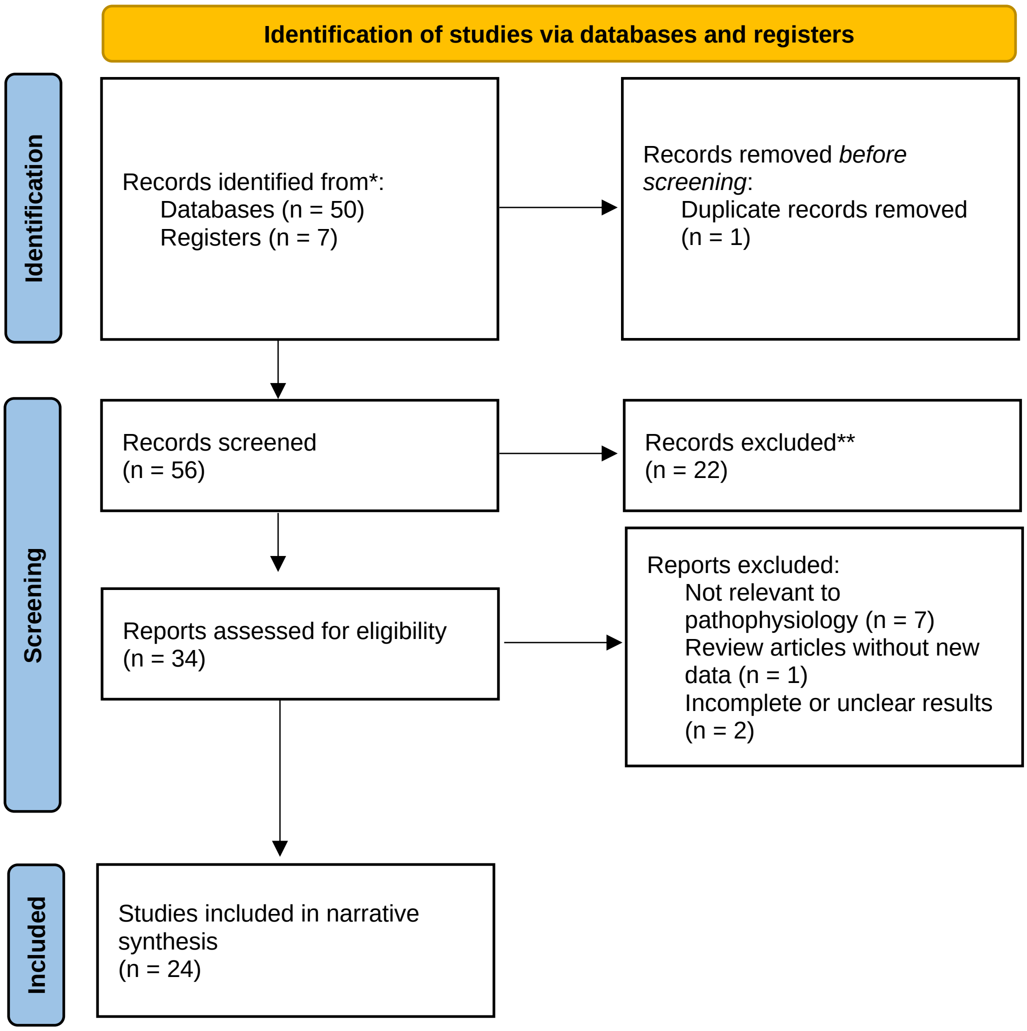

*Consider, if feasible to do so, reporting the number of records identified from each database or register searched (rather than the total number across all databases/registers).

**If automation tools were used, indicate how many records were excluded by a human and how many were excluded by automation tools.
