# Supplementary material for: Microbiota Dysbiosis: A Key Modulator in Preeclampsia Pathogenesis and Its Therapeutic Potential
Source: Microorganisms. 2025 Jan 23;13(2):245. doi: 10.3390/microorganisms13020245 (PMC11857279; doi:10.3390/microorganisms13020245)
Supplement: Supplementary file 1 [file microorganisms-13-00245-s001.zip › Table S1.pdf]

**Table S1.** Search Strategies	
Database	Search Terms
PubMed	(Preeclampsia[MeSH] OR preeclampsia[Title/Abstract] OR hypertensive disorders of pregnancy[Title/Abstract] OR gestational hypertension[Title/Abstract]) AND (Microbiota[MeSH] OR microbiota[Title/Abstract] OR microbial community[Title/Abstract] OR gut microbiome[Title/Abstract] OR vaginal microbiota[Title/Abstract] OR intestinal microbiota[Title/Abstract] OR dysbiosis[Title/Abstract]) AND (Pregnancy[MeSH] OR pregnancy[Title/Abstract] OR pregnant[Title/Abstract] OR gestation[Title/Abstract]) AND (Inflammation[MeSH] OR inflammation[Title/Abstract] OR immune response[Title/Abstract] OR immune system[Title/Abstract] OR immune modulation[Title/Abstract] OR inflammatory response[Title/Abstract])
MEDLINE	(Preeclampsia[MeSH] OR preeclampsia[Title/Abstract] OR hypertensive disorders of pregnancy[Title/Abstract] OR gestational hypertension[Title/Abstract]) AND (Microbiota[MeSH] OR microbiota[Title/Abstract] OR microbial community[Title/Abstract] OR gut microbiome[Title/Abstract] OR vaginal microbiota[Title/Abstract] OR intestinal microbiota[Title/Abstract] OR dysbiosis[Title/Abstract]) AND (Pregnancy[MeSH] OR pregnancy[Title/Abstract] OR pregnant[Title/Abstract] OR gestation[Title/Abstract]) AND (Inflammation[MeSH] OR inflammation[Title/Abstract] OR immune response[Title/Abstract] OR immune system[Title/Abstract] OR immune modulation[Title/Abstract] OR inflammatory response[Title/Abstract])
Google Scholar	"Preeclampsia" OR "hypertensive disorders of pregnancy" OR "gestational hypertension" AND "Microbiota" OR "microbial community" OR "gut microbiome" OR "vaginal microbiota" OR "intestinal microbiota" OR "dysbiosis" AND "Pregnancy" OR "pregnant" OR "gestation" AND "Inflammation" OR "immune response" OR "immune system" OR "immune modulation" OR "inflammatory response"
Scopus	TITLE-ABS-KEY ("Preeclampsia" OR "hypertensive disorders of pregnancy" OR "gestational hypertension") AND TITLE-ABS-KEY ("Microbiota" OR "microbial community" OR "gut microbiome" OR "vaginal microbiota" OR "intestinal microbiota" OR "dysbiosis") AND TITLE-ABS-KEY ("Pregnancy" OR "pregnant" OR "gestation") AND TITLE-ABS-KEY ("Inflammation" OR "immune response" OR "immune system" OR "immune modulation" OR "inflammatory response")
