# Supplementary material for: Microbiota Dysbiosis: A Key Modulator in Preeclampsia Pathogenesis and Its Therapeutic Potential
Source: Microorganisms. 2025 Jan 23;13(2):245. doi: 10.3390/microorganisms13020245 (PMC11857279; doi:10.3390/microorganisms13020245)
Supplement: Supplementary file 1 [file microorganisms-13-00245-s001.zip › Table S2.pdf]

**Table S2.** Summary of included studies					
No.	Title	Authors	Year	Journal	DOI
1	Microbiome Changes in Pregnancy Disorders	Giannella, L., Grelloni, C., Quintili, D., Fiorelli, A., Montironi, R., Alia, S., Delli Carpini, G., Di Giuseppe, J., Vignini, A., & Ciavattini, A.	2023	Antioxidants	DOI: 10.3390/antiox12020463
2	Gut Dysbiosis Promotes Preeclampsia by Regulating Macrophages and Trophoblasts	Jin, J., Gao, L., Zou, X., Zhang, Y., Zheng, Z., Zhang, X., Li, J., Tian, Z., Wang, X., Gu, J., Zhang, C., Wu, T., Wang, Z., & Zhang, Q.	2022	Circulation Research	DOI: 10.1161/CIRCRESAHA.122.320771
3	Landscapes of gut bacterial and fecal metabolic signatures and their relationship in severe preeclampsia	Liu, X., Zeng, X., Li, X., Xin, S., Zhang, F., Liu, F., Zeng, Y., Wu, J., Zou, Y., & Xiong, X.	2024	Journal of translational medicine	DOI: 10.1186/s12967-024-05143-5
4	Gut Microbiota Dysbiosis and Increased Plasma LPS and TMAO Levels in Patients With Preeclampsia	Wang, J., Gu, X., Yang, J., Wei, Y., & Zhao, Y.	2019	Frontiers in cellular and infection microbiology	DOI: 10.3389/fcimb.2019.00409
5	Early-Onset Preeclampsia Is Associated With Gut Microbial Alterations in Antepartum and Postpartum Women.	Lv, L. J., Li, S. H., Li, S. C., Zhong, Z. C., Duan, H. L., Tian, C., Li, H., He, W., Chen, M. C., He, T. W., Wang, Y. N., Zhou, X., Yao, L., & Yin, A. H.	2019	Frontiers in cellular and infection microbiology	DOI: 10.3389/fcimb.2019.00224
6	Gut Micro- and Mycobiota in Preeclampsia: Bacterial Composition Differences Suggest Role in Pathophysiology	Meijer, S., Pasquinelli, E., Renzi, S., Lavasani, S., Nouri, M., Erlandsson, L., Cavalieri, D., & Hansson, S. R.	2023	Biomolecules	DOI: 10.3390/biom13020346
7	The unique immunological and microbial aspects of pregnancy	Mor, G., Aldo, P., & Alvero, A. B.	2017	Nature reviews. Immunology,	DOI: 10.1038/nri.2017.64
8	Microbial Changes during Pregnancy, Birth, and Infancy	Nuriel-Ohayon, M., Neuman, H., & Koren, O.	2016	Frontiers in microbiology	DOI: 10.3389/fmicb.2016.01031
9	Host remodeling of the gut microbiome and metabolic changes during pregnancy	Koren, O., Goodrich, J. K., Cullender, T. C., Spor, A., Laitinen,	2012	Cell	DOI: 10.1016/j.cell.2012.07.008

		K., Bäckhed, H. K., Gonzalez, A., Werner, J. J., Angenent, L. T., Knight, R., Bäckhed, F., Isolauri, E., Salminen, S., & Ley, R. E.			
10	Gut microbiota, low-grade inflammation, and metabolic syndrome	Chassaing, B., & Gewirtz, A. T.	2014	Toxicologic pathology	DOI: 10.1177/0192623313508481
11	[The importance of maternal microbiome in pregnancy]	Záhumenský, J., Hederlingová, J., & Pšenková, P.	2017	Ceska gynekologie	PMID: 28593775
12	Advances in Research on the Relationship between Vaginal Microbiota and Adverse Pregnancy Outcomes and Gynecological Diseases	Zhao, F., Hu, X., & Ying, C.	2023	Microorganisms	DOI: 10.3390/microorganisms11040991
13	Severe preeclampsia is associated with a higher relative abundance of Prevotella bivia in the vaginal microbiota	Lin, C. Y., Lin, C. Y., Yeh, Y. M., Yang, L. Y., Lee, Y. S., Chao, A., Chin, C. Y., Chao, A. S., & Yang, C. Y.	2020	Scientific reports	DOI: 10.1038/s41598-020-75534-3
14	Maternal microbiome in preeclampsia pathophysiology and implications on offspring health	Ishimwe J. A.	2021	Physiological reports	DOI: 10.14814/phy2.14875
15	The placenta harbors a unique microbiome	Aagaard, K., Ma, J., Antony, K. M., Ganu, R., Petrosino, J., & Versalovic, J.	2014	Science translational medicine	DOI: 10.1126/scitranslmed.3008599
16	Assessing the involvement of the placental microbiome and virome in preeclampsia using non coding RNA sequencing	Yoffe, L., Kuperman, A. A., Isakov, O., Haguel, D., Polsky, A. L., Farberov, L., Pillar, N., Gurevich, V., Haviv, I., & Shomron, N.	2021	Journal of perinatal medicine	DOI: 10.1515/jpm-2021-0006
17	Gut dysbiosis induces the development of pre-eclampsia through bacterial translocation	Chen, X., Li, P., Liu, M., Zheng, H., He, Y., Chen, M. X., Tang, W., Yue, X., Huang, Y., Zhuang, L., Wang, Z., Zhong, M., Ke, G., Hu, H., Feng, Y., Chen, Y., Yu, Y.,	2020	Gut	DOI: 10.1136/gutjnl-2019-319101

		Zhou, H., & Huang, L.			
18	Gestational diabetes is associated with change in the gut microbiota composition in third trimester of pregnancy and postpartum	Crusell, M. K. W., Hansen, T. H., Nielsen, T., Allin, K. H., Rühlemann, M. C., Damm, P., Vestergaard, H., Rørbye, C., Jørgensen, N. R., Christiansen, O. B., Heinsen, F. A., Franke, A., Hansen, T., Lauenborg, J., & Pedersen, O.	2018	Microbiome	DOI: 10.1186/s40168-018-0472-x
19	Gut Microbiota in Cardiovascular Health and Disease	Tang, W. H., Kitai, T., & Hazen, S. L.	2017	Circulation research	DOI: 10.1161/CIRCRESAHA.117.309715
20	Regulation of the stress response by the gut microbiota: implications for psychoneuroendocrinology	Dinan, T. G., & Cryan, J. F.	2012	Psychoneuroendocrinology	DOI: 10.1016/j.psyneuen.2012.03.007
21	Connections Between the Gut Microbiome and Metabolic Hormones in Early Pregnancy in Overweight and Obese Women	Gomez-Arango, L. F., Barrett, H. L., McIntyre, H. D., Callaway, L. K., Morrison, M., Dekker Nitert, M., & SPRING Trial Group	2016	Diabetes	DOI: 10.2337/db16-0278
22	Perinatal outcomes of prenatal probiotic and prebiotic administration: an integrative review	VandeVusse, L., Hanson, L., & Safdar, N.	2013	Journal of perinatal & neonatal nursing	DOI: 10.1097/JPN.0b013e3182a1e15d
23	The effect of dietary fiber supplement on prevention of gestational diabetes mellitus in women with pre-pregnancy overweight/obesity: A randomized controlled trial	Zhang DY, Cheng DC, Cao YN, Su Y, Chen L, Liu WY, et al.	2022	Front Pharmacol	DOI: 10.3389/fphar.2022.922015
24	Dietary factors that affect the risk of pre-eclampsia	Perry A, Stephanou A, Rayman MP	2022	BMJ Nutr Prev Health	DOI: 10.1136/bmjnph-2021-000399
